# Assessment of Pesticide Residues and Dietary Risks in Ginseng from Northeastern China

**DOI:** 10.3390/foods14081381

**Published:** 2025-04-17

**Authors:** Xuanwei Xu, Min Zhang, Xinxin Meng, Ying Chen, Xu Leng, Shuang Liang, Dan Zhao

**Affiliations:** 1 Ginseng and Antler Products Testing Center of the Ministry of Agricultural and Rural Affairs, Jilin Agricultural University, Changchun 130118, China; xuanweix@jlau.edu.cn (X.X.); minz@jlau.edu.cn (M.Z.); mengxinxin@jlau.edu.cn (X.M.); yingc@jlau.edu.cn (Y.C.); 2 College of Plant Protection, Jilin Agricultural University, Changchun 130118, China; leng06132023@163.com (X.L.); liang84shuang@163.com (S.L.)

**Keywords:** ginseng, dietary risk assessment, food safety, phoxim, chlorpyrifos

## Abstract

Several challenges persist in China’s ginseng industry. Phoxim, chlorpyrifos, quintozene—unregistered pesticides primarily used as soil insecticides and fungicides—may pose high dietary risks. This study performed a thorough screening of potential pesticides used in Chinese ginseng cultivation, evaluated the long-term dietary risks for the ginseng-consuming group, and used the primary risk factors for ginseng in China and South Korea to compare the two nations’ pesticide usage scenarios. From 2020 to 2022, 325 pesticides and related compounds were screened in 15 major ginseng-producing counties and 3 commercial markets in Northeast China, and 39 pesticides and 3 metabolites were identified using gas chromatography–tandem mass spectrometry and liquid chromatography–tandem mass spectrometry, including allylmorph, pyrimethanil, pyraclostrobin, and other contaminants in Northeast China. Acute and chronic dietary risk assessment was performed using 0.009 kg as the maximum daily intake and 0.005 kg as the average daily intake, with adults as the exposed population. Based on these findings and reliable toxicological data, acute and chronic dietary risk quotients for ginseng were assessed, considering dietary intake and population exposure. The results indicate that ginseng products are generally safe and manageable, with acute and long-term dietary risks remaining within acceptable limits.

## 1. Introduction

Ginseng, a perennial plant of the *Araliaceae* family and the *Panax* genus, is highly valued in traditional medicine, particularly in East Asia, for its medicinal properties [1,2,3]. The primary medicinal component of ginseng is its root, which is significant for often resembling the human body [4]. These roots contain ginsenosides, the active compounds believed to contribute to its health benefits. Traditionally, ginseng has been used to improve physical endurance, support cognitive function, strengthen the immune system, and reduce stress [5,6]. It is also thought to display anti-inflammatory and antioxidant properties. Modern research continues to explore its potential role in managing conditions such as diabetes, cancer, and cardiovascular diseases [7,8].

Ginseng is both a food and a traditional Chinese medicine, sharing the same origin [9]. With the continuous expansion of the ginseng industry and growing public awareness of health care, an increasing number of ginseng products have entered the market, leading to a larger consumer base. According to the *Pharmacopoeia of the People’s Republic of China*, ginseng consumption is not recommended for children [10]. In China, the primary consumers of ginseng are middle-aged and elderly individuals, and adults with weakened immunity. Some consumers habitually take ginseng or its products long-term, necessitating higher quality standards. However, long-term consumption of high-risk products may pose varying degrees of chronic toxicity, including but not limited to carcinogenic effects, to consumers. The current monitoring of pesticide residues in ginseng does not fully encompass the actual pesticides used. Given this limitation, a comprehensive risk assessment is essential to evaluate the quality of ginseng products in China.

Different countries and regions have established varying pesticide residue limits for ginseng. GB 2763-2021 [11] includes only 72 pesticide residue limits that apply to all ginseng products, and testing methods are not specified for 21 of these substances [11]. The European Union (EU) has set 476 limits, including 74 pesticides banned within the EU, with 222 being unique pesticides. Canada has established 36 residue limits for ginseng, while Japan has set 300 standards [12]. South Korea has defined 123 limits for fresh ginseng, 103 for dried ginseng, and 45 for red ginseng [13]. The International Codex Alimentarius Commission (CAC) has set 15 standards for ginseng, dried ginseng, and ginseng extracts, covering eight pesticides [14,15]. Moreover, pesticide residue limits for ginseng differ across countries. For instance, China’s limit for pyrimethanil is stricter than Japan’s but less stringent than those of the EU, CAC, South Korea, and Canada. However, its limit for phenyl ether metronidazole matches South Korea’s, is stricter than Japan’s and the EU’s, but less stringent than the CAC’s. Standards play an important role in ensuring the sustainable development and industrialization of the ginseng industry. They serve as fundamental technical tools for improving product quality and boosting industrial competitiveness, making them essential for advancing ginseng production, regulating the market, safeguarding consumer rights, and expanding exports [16]. However, the current standardization system in China falls short of providing comprehensive oversight of ginseng product quality. In 2019, Wang et al. assessed the risks associated with 80 ginseng samples and their analysis identified 25 pesticides (18 fungicides, 6 insecticides, and 1 herbicide), followed by acute and chronic risk assessments and a comprehensive risk ranking. The findings revealed that unregistered pesticides posed the highest risk among the six most concerning pollutants in ginseng [17]. Sun et al. [18] conducted a risk assessment of azoxystrobin and metalaxyl in ginseng and three other Chinese herbal medicines, concluding that their residues in ginseng did not pose acute or chronic dietary risks. Relatively few studies in China have assessed the risks associated with ginseng products. In South Korea, Kim Jung-ho et al. analyzed and evaluated pesticide residues in fresh ginseng and red ginseng concentrate [19]. This study aims to comprehensively assess contaminants, including non-standardized and unregulated pesticides, identify potential safety concerns, and evaluate the quality and safety risks of ginseng. The findings will help implement timely and effective measures to safeguard public health. Since 29 August 2012, the Chinese Ministry of Health has classified artificially cultivated ginseng as a new resource food [20], formally integrating it into the food sector.

This study aims to assess the potential dietary risks associated with ginseng in China by employing a screening method to identify risk factors and conduct a comprehensive dietary risk assessment. The analysis will focus on major ginseng cultivation bases, including large-scale producers, industry cooperatives, and individual growers, across Jilin, Heilongjiang, and Liaoning provinces, along with key ginseng trading markets in Northeast China.

## 2. Materials and Methods

### 2.1. Sample Collection

- Assessment Area

The assessment covered ginseng planting bases across 15 ginseng-producing counties, including Tieli and Baoqing in Heilongjiang; Fusong, Changbai, Linjiang, Jingyu, Hunchun, Dunhua, Wangqing, Antu, Ji’an, Tonghua, Jiaohe, and Huadian in Jilin; and Huanren Manchurian Autonomous County in Liaoning. This study encompassed large-scale ginseng cultivation enterprises, ginseng cooperatives, and individual growers. Samples were also collected from ginseng wholesale markets in Heilongjiang, Jilin, and Liaoning. The specific sampling locations and sample numbers are detailed in Table 1.

Samples were collected during three periods: from 6 August to 12 September 2020; 13 August to 16 September 2021; and 3 August to 12 September 2022.

Sample collection method: Fresh ginseng samples were obtained through on-site digging at ginseng planting bases and direct purchases from ginseng markets. The collected samples consisted of fresh ginseng aged 3 to 6 years. Each sample included at least eight individual ginseng plants, with a minimum total weight of 500 g. To prevent duplication, only one sample was collected per year from each ginseng planting base.

Number of samples: A total of 325 fresh ginseng samples were collected, with 133 samples in 2020, 100 in 2021, and 92 in 2022. The detailed sample distribution is provided in Table 1.

### 2.2. Development of Detection Method

#### 2.2.1. Sample Preparation

Ginseng samples were homogenized using a tissue masher, dried at 60 °C, and further ground using a Chinese medicine crusher. The resulting powder was sieved through a 60-mesh sieve and stored in sealed bags at a freezing temperature of −18 °C for subsequent analysis.

#### 2.2.2. Extraction of Samples

Approximately 3 g of ginseng powder (accurate to 0.01 g) was weighed into 50 mL plastic centrifuge tubes. For quality control, spiked samples were allowed to stand for 10 min after the addition of standards. Subsequently, 10 mL of a 1% acetic acid solution (*v*/*v* = 1/99) was added, and the mixture was shaken and soaked for 30 min using SIO-6512 QuEChERS Automatic Sample Preparation System. Following this step, 12 mL of acetonitrile was added, and the mixture was vigorously shaken for 5 min. To the mixture, 6 g of anhydrous magnesium sulfate, 1.5 g of sodium acetate, and 1 ceramic homogenizer were added, followed by another 5 min of vigorous shaking. The sample was then centrifuged at 4500 rpm for 5 min. A 9 mL aliquot of the supernatant was transferred to a 15 mL polypropylene (PP) centrifuge tube containing 900 mg of anhydrous magnesium sulfate and 150 mg of PSA as adsorbents for purification.

#### 2.2.3. Sample Purification

The 15 mL centrifuge tube was shaken vigorously for 1 min and centrifuged at 5000 rpm for 5 min. A 4 mL portion of the supernatant was transferred to a 10 mL graduated centrifuge tube and evaporated to dryness under a nitrogen stream in a 60 °C water bath. The residue was reconstituted in ethyl acetate containing 0.1 μg/mL triphenyl phosphate (internal standard) and adjusted to a final volume of 1 mL. The solution was filtered through a 0.22 μm membrane and analyzed by gas chromatography–tandem mass spectrometry (GC-MS/MS).

For ultra-high-performance liquid chromatography–tandem mass spectrometry (UHPLC-MS/MS) analysis, an additional 2 mL of the supernatant was transferred to a 5 mL graduated centrifuge tube, evaporated to dryness under nitrogen, and reconstituted in 0.5 mL of methanol. The solution was then diluted with a 5 mmol/L ammonium formate aqueous solution containing 0.1% formic acid, adjusted to a final volume of 1 mL, filtered through a 0.22 μm membrane, and subjected to UHPLC-MS/MS analysis.

#### 2.2.4. Determination of Samples

##### Gas Chromatography–Tandem Mass Spectrometry (GC-MS/MS) Analysis

GC-MS/MS Conditions

Inlet temperature: 260 °C; column: DB-1701 quartz capillary column (30 m × 0.32 mm × 0.25 µm, (Agilent, Santa Clara, CA, USA)), column temperature: $100\;{}^{\circ}C\;(1\;\min)\;\overset{\Delta 10\;{}^{\circ}C/\min}{\to }180\;{}^{\circ}C\;(5\;\min)\;\overset{\Delta 20\;{}^{\circ}C/\min}{\to }285\;{}^{\circ}C\;(8\;\min)$; carrier gas and flow rate: high-purity helium at 1.2 mL/min; injection volume: 1.0 µL; ion source temperature: 300 °C; chromatography–mass spectrometry interface temperature: 280 °C; ionization mode: electron ionization (EI), positive ion mode; scanning mode: multiple reaction monitoring (MRM); injection mode: spitless injection; quantification method: internal standard method. The detection parameters and recoveries for GC-MS/MS are summarized in Supplementary Materials Table S1.

##### Ultra-High-Performance Liquid Chromatography–Tandem Mass Spectrometry (UHPLC-MS/MS) Analysis

UHPLC Conditions

Column: XB-C_18_ column (100 mm × 2.1 mm, 2.6 µm, Philmont, Guangzhou, China); mobile phase: A: 5 mmol/L ammonium formate aqueous solution containing 0.1% formic acid; B: methanol; gradient elution program: 0–6 min: 5–80% B; 6–6.1 min: 80–95% B; 6.1–8 min: 95% B; 8–8.1 min: 95–5% B; 8.1–11 min: 5% B; column temperature: 40 °C; injection volume: 5.0 µL; quantification method: external standard method

MS/MS Conditions

Ionization mode: electrospray ionization (ESI); ionization polarity: positive mode; ionization voltage: 5500 V; nebulizer gas pressure: 0.38 MPa; auxiliary heating gas pressure: 0.41 MPa; curtain gas pressure: 0.2 MPa; scanning mode: multiple reaction monitoring (MRM); monitoring ion pairs, collision energies, and cone voltages: Supplementary Materials Table S2 for details.

#### 2.2.5. Limit of Detection and Limit of Quantification

In this study, the limit of detection and limit of quantification were evaluated for 215 pesticides. The limits of detection (LODs) for all pesticides ranged from 0.003 μg/kg to 3.2 μg/kg, while the limits of quantification (LOQs) ranged from 0.01 μg/kg to 10 μg/kg. These LOQs were in accordance with or better than the national minimum limit value of 10 μg/kg for all pesticide residues. This result indicates that the self-constructed method can effectively meet the stringent requirements for the detection of pesticide residues.

#### 2.2.6. Accuracy and Precision

The average recoveries of the pesticides ranged from 36.7% to 144.5%, with relative standard deviation (RSD) values between 0.1% and 25.5%. It was shown that 197 target pesticides had recoveries in the range of 70% to 130%, which met the requirements for quantitative analysis. The remaining targets were suitable for qualitative analysis and screening. In addition, the standard curves of all 215 targets showed a high degree of linearity, with linear correlation coefficients exceeding 0.99. The results indicate that the methodology adopted in this study is valid and reliable for the analysis of 197 target pesticide residues.

### 2.3. Risk Assessment of Ginseng Product Quality

Acute and chronic dietary risk assessments, along with risk factor rankings for contaminants detected in ginseng from 2020 to 2022, were performed. The exposure assessment combined contaminant levels in food with dietary consumption data from the exposed population. Statistical methods were then used to estimate dietary exposure. This estimate determined the actual or expected human exposure to hazardous factors.

Mathematical simulation models, typically based on point and probabilistic assessments, are commonly used for exposure evaluation. In this study, a point assessment method was applied to separately calculate acute and chronic ginseng exposure levels. Point assessment is a straightforward and widely used approach for identifying high-risk contaminants in food. Its principle prioritizes the protection of the majority of the population, making it the most suitable method for exposure screening in risk assessments.

#### 2.3.1. Acute Dietary Risk Assessment

The U.S. Environmental Protection Agency (EPA) point assessment model [15] was used in this study, and the exposure models for the acute and chronic point assessments were calculated using the following equations, respectively:

(1) $$e_{i,p}=\frac{\sum_{a=1}^{p}c_{i}\times q_{p}}{bw_{i}},$$

(2) $$\%ARfD=\frac{e_{i,p}}{ARfD}\times 100\%$$

where e_i,p_ is the acute dietary exposure of the corresponding pesticide in each sample in mg-d^−1^-kg^−1^bw); c_i_ is the maximum amount of pesticide residue in the consumed ginseng sample (mg/kg); q_p_ is the 97.5th percentile value of the maximum daily consumption of ginseng containing the corresponding pesticide (kg·d^−1^), which is not recommended by the latest 2020 edition of the *Pharmacopoeia of the People’s Republic of China* and the *2022 Dietary Guidelines for Chinese Residents* (DGCR). Therefore, in this study, the maximum daily ginseng consumption for the exposed population was set at 0.009 kg, based on the daily dosage range of 3–9 g specified in the 2015 edition of the *Pharmacopoeia of China*. *bwi* represents the body weight of exposed individuals (kg), which was set at an average of 60 kg for adults, as children are not permitted to consume ginseng.

The acute ingestion risk was assessed using *e*_*i*_, with the risk level determined by its ratio to the acute reference dose (ARfD). If the ratio is ≤100%, the hazard is considered to pose an acceptable risk. However, if the ratio is ≥100%, the risk exceeds acceptable limits, necessitating appropriate risk management measures.

#### 2.3.2. Chronic Dietary Risk Assessment

The chronic dietary risk assessment also employed the U.S. EPA point assessment model [11], with the exposure model for the chronic point assessment calculated using the following equations.

(3) $$EXPC=\frac{I\times \bar{C}}{bw_{i}},$$

(4) $$\%ADI=\frac{EXPC}{ADI}\times 100\%$$

where EXPc represents the chronic dietary exposure, while I denotes the average daily ginseng consumption, which is 0.005 kg according to the *Chinese Pharmacopoeia*. b_wi_ refers to the average body weight of the exposed population (kg), set at 60 kg. In domestic risk assessments, the ratio of EXPc to ADI is commonly used to evaluate chronic intake risk. If the ratio is ≤100%, the risk posed by the hazardous substance is considered acceptable. However, if the ratio is ≥100%, the risk exceeds acceptable limits, requiring appropriate risk management measures.

## 3. Results

### 3.1. Pesticide Detection and Distribution in Ginseng Cultivation

To improve the statistical significance of the risk assessment, only pesticides detected three or more times were included in the analysis. This approach excluded pesticides with extremely low detection frequencies, ensuring more reliable and representative results. Over the three-year assessment, 39 pesticides and 3 pesticide metabolites were identified. Fungicides comprised the largest category, with 28 pesticides accounting for 71.8% of the total, alongside 7 insecticides and 4 herbicides. These findings highlighted the predominant pesticide types used in ginseng cultivation and their relative proportions. In terms of both the number of collected samples and the types of detected pollutants, this assessment was the largest conducted to date on Chinese ginseng. Notably, mefentrifluconazole, thifluzamide, and several other pesticides were detected for the first time. The detection results also provided insights into pesticide usage patterns [21,22].

Among the detected pesticides, 21 were legally registered for use in ginseng, accounting for 53.8% of the total. The nine most frequently detected pesticides were all low-toxicity fungicides, resulting in a fungicide detection frequency of 89.3%. In comparison, insecticides and herbicides had detection frequencies of 9.7% and 1.0%, respectively. Table 2 presents the specific pesticides detected, along with their detection frequencies and quantities.

### 3.2. Annual Risk Screening Results

In 2020, 35 pesticides (metabolites counted as parent compounds) were detected in 133 samples, while 31 pesticides were found in 100 samples in 2021 and 32 pesticides in 92 samples in 2022. Each year, the 20 most frequently detected pesticides remained predominant. The pesticides with the highest detection rates were dimethomorph (74.8%) in 2020, pyrimethanil (58%) in 2021, and pyraclostrobin (54.8%) in 2022.

### 3.3. Discussion of Screening Results

The screening results indicate that pesticide use in ginseng cultivation in China primarily targets disease control, followed by underground pest management and pre-seedling weed control. Fungicide use was generally standardized, with most highly detected fungicides being registered low-toxicity low-residue varieties, except for quintozene. By comparison, insecticide use was highly irregular, with most detected insecticides being unregistered, except for cyhalothrin, thiamethoxam, and clothianidin. Some detected insecticides also included medium to highly toxic pesticides, such as carbofuran, which are explicitly banned in Chinese herbal medicine and agricultural products.

**Figure 1:**
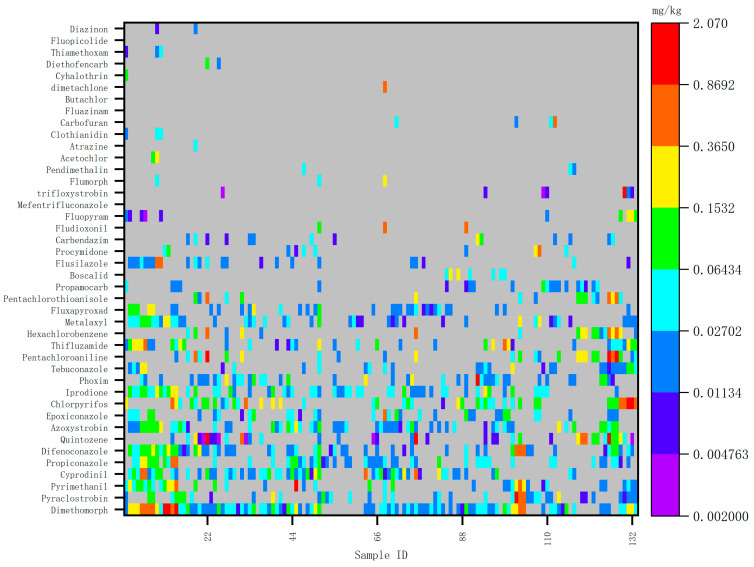
Detected values of risk screening samples in 2020.

**Figure 2:**
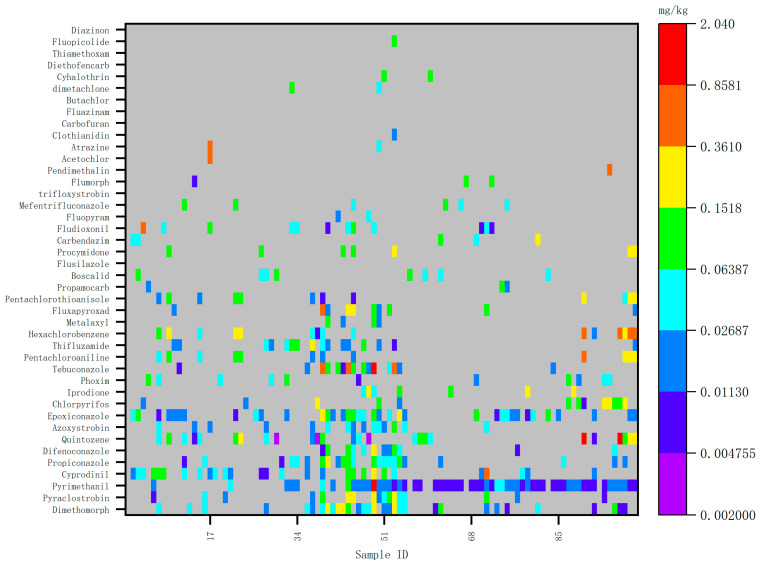
Detected values of risk screening samples in 2021.

**Figure 3:**
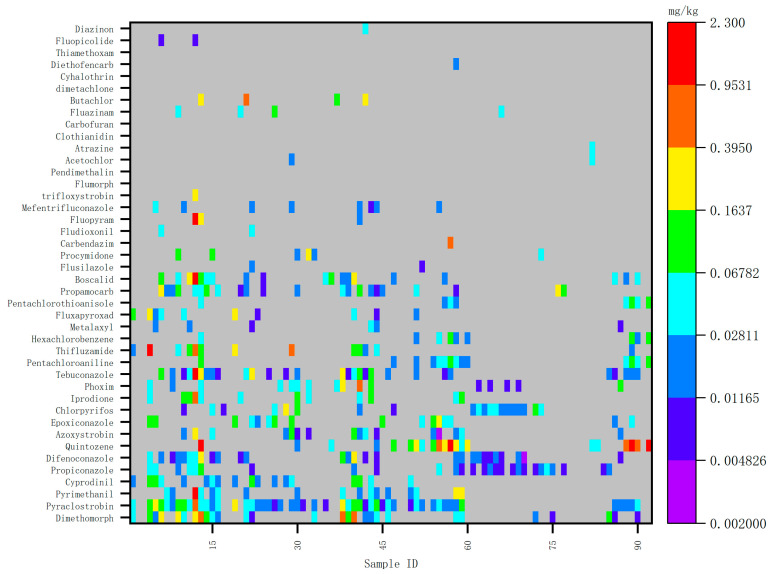
Detected values of risk screening samples in 2022.

The types and frequency of pesticide detection in ginseng cultivation in China have remained generally consistent in recent years, though some variability exists. New pesticides are annually introduced, indicating that the selection of pesticides is not fixed, and their usage frequency varies significantly. Heat maps illustrating pesticide detection values for 2020, 2021, and 2022 are presented in Figure 1, Figure 2 and Figure 3.

As one of the leading producers of ginseng globally, South Korea possesses relatively advanced technology for ginseng cultivation. This paper aims to compare the results of risk assessments with those from earlier evaluations conducted in South Korea, thereby assessing the differences in pesticide usage practices and the quality of ginseng products between the two countries. Song et al. [23] analyzed pesticide residues in ginseng with and without rhizomes from eight production bases in Cheongju, South Korea. Samples were collected 10 days before harvest for assessment. Their results indicate similarities in fungicide use between China and Korea but significant differences in insecticide application [24]. In total, 40 pesticides (29 fungicides and 11 insecticides) were detected in South Korea, with fungicides accounting for 72.5% of the total. Meanwhile, 19 fungicides were found in both China and South Korea, comprising approximately 50% of the total pesticides detected in both regions. Overall, fungicides constituted nearly 70% of pesticide use in both countries.

A total of 11 insecticides were further detected in South Korea, accounting for 27.5% of all pesticides, a significantly higher number than in China. Apart from thiamethoxam and clothianidin, the insecticides used in the two countries were entirely different. In South Korea, the most commonly used pesticides were cypermethrin (39.6%), dinotefuran (25%), and tolclofos-methyl (22.9%), all of which are registered pesticides in the country but were not detected in China. The primary insecticides detected in China included chlorpyrifos (23.7%), phoxim (21.5%), and clothianidin (1.2%), with chlorpyrifos and phoxim being unregistered pesticides. This difference may mainly depend on different policies, cultivation patterns, and pesticide application habits. Up until now, a total of 61 pesticide formulations (45 active ingredients) have been registered for ginseng in China. In contrast, there are 239 pesticide formulations and 127 active ingredients registered in South Korea. South Korea has more flexibility in pesticide selection, especially for insecticides. Secondly, field-grown ginseng is dominant in South Korea, while forest-grown ginseng accounts for a higher proportion in China [13,25].

**Figure 4:**
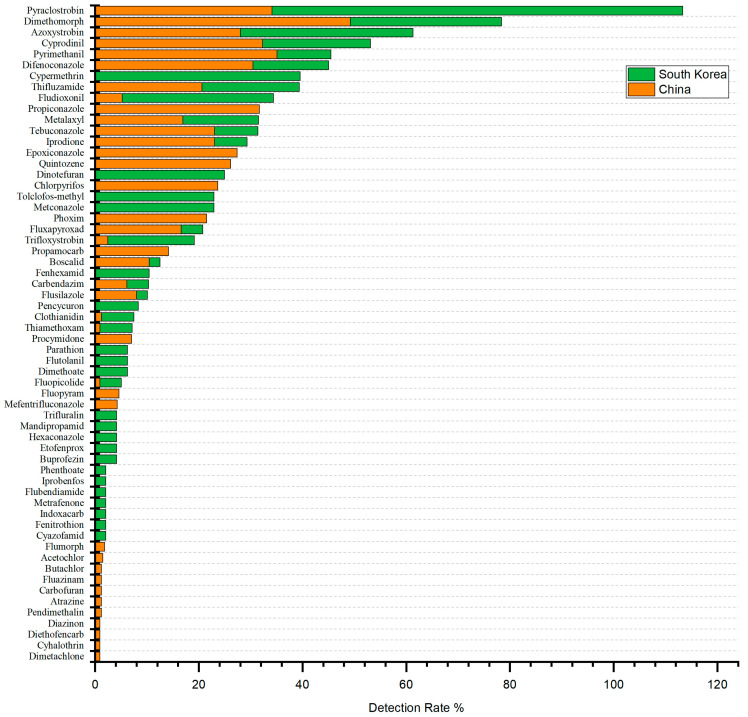
Comparison of detection rates of ginseng pesticides in China and Korea.

Both China and South Korea displayed irregularities in pesticide use. However, the predominant pesticide in South Korea, cypermethrin, is less toxic than chlorpyrifos, which is more commonly used in China [26,27,28]. In China, restricted pesticides such as carbofuran and chlorpyrifos were detected, whereas South Korea identified highly toxic pesticides like dimethoate and parathion, which are banned in China. These findings suggest that the types and quantities of insecticides registered in ginseng in China do not yet meet the actual needs. There are currently 45 pesticides registered for use on ginseng in China, of which there are only 3 insecticides, cyhalothrin, thiamethoxam and clothianidin, and none of the 3 insecticides can effectively control underground pests, which makes pesticides such as carbofuran and chlorpyrifos a possible choice [29]. This is also related to the lack of knowledge of growers and the higher prices of the latest registered pesticides. The specific pesticides detected in ginseng from China and South Korea, along with their detection rates, are presented in Figure 4.

### 3.4. Acute Dietary Risk Assessment Analysis

**Figure 5:**
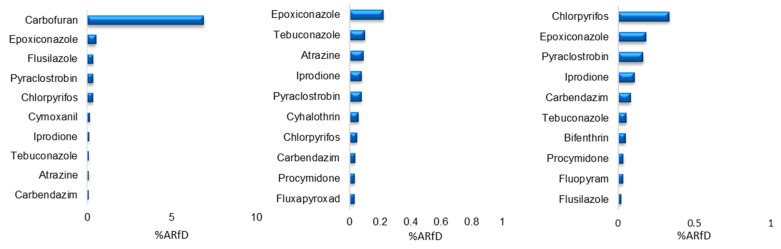
Ranking of acute dietary risks of ginseng samples.

The toxicological data in this study were obtained from official sources, including the Food and Agriculture Organization of the United Nations (FAO), the Joint Meeting on Pesticide Residues (JMPR) of the World Health Organization (WHO), the EU pesticide database, the national standard of the People’s Republic of China, the national food safety standard GB 2763-2021 [11], and Japan’s positive list. A total of 33 pesticides were identified for toxicological assessment, as detailed in Table 3. Due to the absence of an ARfD for some pesticides or official statements indicating that an ARfD is unnecessary, these pesticides were excluded from risk calculations. The results of the 10 highest acute dietary risk factors are presented in Figure 5.

In 2020, the ginseng sample with the highest acute dietary risk was associated with carbofuran (0.46 mg/kg), which had an acute risk quotient (ARQ) of 6.9%. This value is well below the 100% threshold, indicating no acute dietary risk. Carbofuran is primarily used as a soil treatment in the spring to protect young ginseng roots from underground pests during sowing and transplanting. The overall acute dietary risk for ginseng in 2021 was lower than in 2020, with epoxiconazole having the highest risk quotient at 0.22%, which remains far below the 100% acute alert level. In 2022, chlorpyrifos posed the highest acute dietary risk, with a risk quotient of 0.34%, still well below the 100% threshold. The second highest risk factor was epoxiconazole.

### 3.5. Chronic Dietary Risk Assessment Analysis

**Figure 6:**
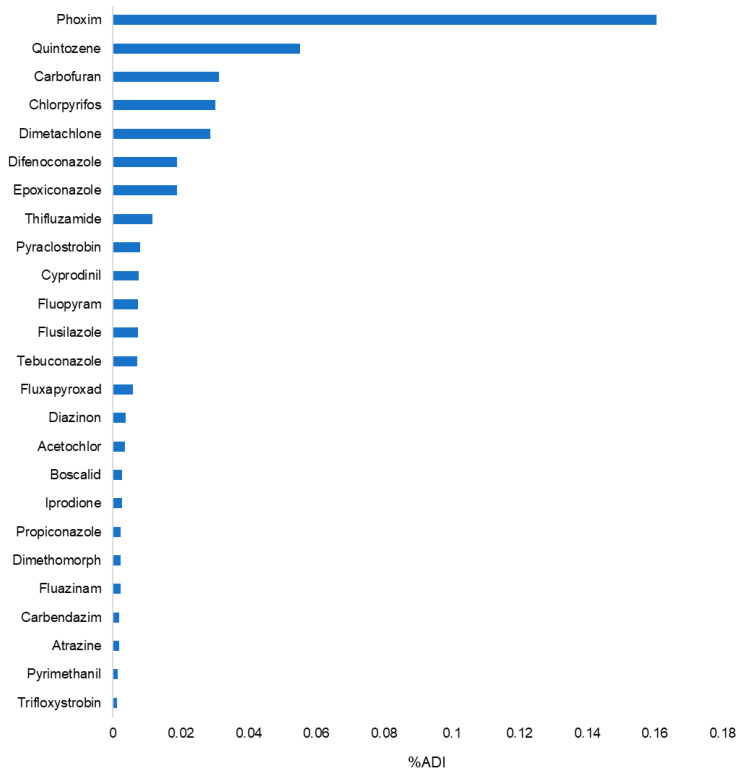
Ranking of chronic diets in 3-year risk assessment.

The GB 2763-2021 [11] standard for quintozene residues does not account for pentachloroaniline, methyl pentachlorophenyl sulfide, or hexachlorobenzene, as residues of these three metabolites or impurities are not considered quintozene. The chronic toxicity risk was assessed and ranked accordingly, with the specific results presented in Figure 6.

As shown in Figure 6, phoxim had the highest chronic dietary risk in ginseng for three consecutive years, with a chronic risk quotient of 0.16%, which remains well below the 100% alert level. The three-year risk assessment data in this study indicate that the primary chronic risk in ginseng from China stems from unregistered pesticides, including phoxim, quintozene, carbofuran, and chlorpyrifos—all of which are used for soil treatment. Overall, the findings suggest that ginseng products from Northeast China are of good quality and do not pose a chronic dietary risk.

## 4. Discussion

### 4.1. Risk Factor Screening

The screening results indicate that pesticide use in ginseng cultivation in China primarily targets disease prevention and control, followed by the management of underground pests and pre-seedling weeding. Fungicide use is generally standardized, with most high-detection-rate fungicides—except for quintozene—being registered, low-toxicity, and low-residue varieties. While the types and detection frequencies of pesticides used in ginseng cultivation have remained largely consistent over the years, there is some variability, with new pesticides appearing annually. This suggests that pesticide use is not fixed and varies in frequency.

### 4.2. Dietary Risk Assessment

The 2019 ginseng quality and safety risk assessment conducted in Korea did not provide specific test values for calculating the acute dietary risk of ginseng in the Cheongju region. However, the report suggests that the acute dietary risk of ginseng in South Korea is comparable to that in China and does not pose a significant concern. Nonetheless, the presence of highly toxic pesticides remains a key issue. In China, the major dietary risk factors for ginseng were identified as phoxim, quintozene, carbofuran, and chlorpyrifos, none of which were detected in the Korean risk assessment. This discrepancy may become a major barrier to the export of Chinese ginseng to Korea. Analysis of the test results indicates that, while carbofuran is used occasionally, the other three pesticides are more commonly applied. Moving forward, the development of efficient, low-toxicity, and low-residue alternatives for soil sterilization and insect control in ginseng cultivation is crucial for reducing its dietary risk in China.

## 5. Conclusions

In conclusion, this study analyzed pesticide residues in ginseng over three years, revealing that fungicides were the most frequently detected (89.3%), while insecticides and herbicides were less common. The findings indicate standardized fungicide use but highlight unregulated insecticide applications, including banned substances. Detection trends remained stable, though new pesticides emerged, differing from patterns observed in South Korea. Stricter regulations on insecticides in China are necessary to improve food safety and compliance. Continuous monitoring and regulatory enforcement will help mitigate health and environmental risks.

This study carried out a three-year pesticide residue risk assessment of 15 major ginseng production areas in the northeastern region of China. This represents the most thorough and methodical assessment of ginseng pesticide residue risk in China since it was documented in the literature and it accounts for the low-risk screening of ginseng in China caused by the lack of standards for ginseng pesticide residue detection and limits. The dietary risk of 325 ginseng samples was calculated by the acute and chronic dietary risk formula, and the results showed that the acute dietary risk of carbofuran and chlorpyrifos was the highest, ranging from 0.34% to 6.9%; and the chronic dietary risk of phoxim, quintozene, and chlorpyrifos was the highest, ranging from 0.038% to 0.47%. The acute and chronic risk values of the above pesticides were much less than the alert value of 100%, indicating that both acute and chronic dietary risks of ginseng cultivated in the main ginseng-producing areas in China are within acceptable limits, but, at the same time, it warns that the irrational use of pesticides in ginseng cultivation in China is relatively common, in which the potential dietary risks mainly originate from insecticides for the control of subterranean pests and soil bactericides, such as phoxim, chlorpyrifos, and quintozene.

## Figures and Tables

**Table 1:** Sampling location and quantity.

Sampling Link	Place of Origin or Sampling	Sampling Area	Number of Samples (Piece)			Total (Piece)
			2020	2021	2022	
Production base	Jilin Province	Fusong	10	5	8	275
		Changbai	5	5	4	
		Linjiang	5	5	4	
		Jingyu	5	10	6	
		Huichun	5	5	5	
		Wangqing	10	5	5	
		Dunhua	8	10	5	
		Antu	5	5	5	
		Ji’an	10	5	5	
		Tonghua	5	5	5	
		Huadian	5	5	5	
		Jiaohe	5	5	5	
	Heilongjiang Province	Tieli	10	5	5	
		Baoqing	5	5	5	
	Liaoning Province	Huanren	10	5	5	
Wholesale market	Jilin Province	Fusong	10	5	5	50
	Liaoning Province	Huanren	10	5	5	
	Heilongjiang Province	Yichun	10	5	5	
Sample count			133	100	92	325

**Table 2:** Detected pesticides, quantities, and residual levels.

No.	Pesticide	Total Detection Times	Recovery	Detection Times			Detectable Value	Mean Value
			%	2020	2021	2022	mg/kg	mg/kg
1	dimethomorph	160	102.4	101	31	28	0.006~1.12	0.11
2	pyrimethanil	114	74.4	39	58	17	0.0067~1.51	0.085
3	pyraclostrobin	111	87.2	43	17	51	0.0075~0.93	0.08
4	cyprodinil	105	77.1	59	28	18	0.007~0.75	0.08
5	propiconazole	103	94.4	53	23	27	0.005~0.82	0.057
6	difenoconazole	99	90.9	59	12	28	0.0032~0.84	0.07
7	azoxystrobin	91	109.2	60	15	16	0.0048~0.38	0.051
8	epoxiconazole	89	102.7	36	37	16	0.0072~0.82	0.061
9	quintozene	85	87.4	39	25	21	0.0033~2.2	0.25
10	chlorpyrifos	77	84.3	44	13	20	0.0056~1.94	0.15
11	iprodione	75	125.1	56	7	12	0.015~0.43	0.074
12	tebuconazole	75	97.4	31	15	29	0.0075~2.04	0.11
13	phoxim	70	90.7	43	11	16	0.0066~1.7	0.082
14	thifluzamide	67	111.4	38	16	13	0.0093~1.75	0.13
15	metalaxyl	55	117.1	45	4	6	0.0077~0.28	0.044
16	fluxapyroxad	54	111.0	34	10	10	0.0084~0.64	0.074
17	pentachloroaniline	53	76.3	27	12	14	0.013~1.26	0.18
18	propamocarb	46	86.6	21	3	22	0.0072~0.24	0.044
19	hexachlorobenzene	44	66.0	20	15	9	0.0055~0.83	0.18
20	methyl-pentachlorophenyl sulfide	37	74.3	17	12	8	0.0065~0.76	0.12
21	boscalid	34	98.5	7	8	19	0.0059~1.26	0.11
22	flusilazole	26	97.4	24	0	2	0.0079~0.47	0.054
23	procymidone	23	97.1	10	7	6	0.011~0.39	0.11
24	carbendazim	20	80.8	14	5	1	0.01~0.48	0.082
25	fludioxonil	17	91.9	3	12	2	0.0094~0.71	0.13
26	fluopyram	15	110.3	10	2	3	0.0039~1.07	0.15
27	mefentrifluconazole	14	127.7	0	6	8	0.011~0.11	0.038
28	trifloxystrobin	8	98.2	7	0	1	0.0037~1	0.16
29	flumorph	6	89.8	3	3	0	0.0081~0.16	0.078
30	acetochlor	5	100.6	2	1	2	0.021~0.37	0.16
31	pendimethalin	4	78.9	3	1	0	0.024~0.61	0.18
32	atrazine	4	84.2	1	2	1	0.036~0.6	0.19
33	clothianidin	4	102.1	3	1	0	0.02~0.046	0.032
34	carbofuran	4	108.4	4	0	0	0.019~0.46	0.14
35	fluazinam	4	92.3	0	0	4	0.034~0.075	0.054
36	butachlor	4	119.1	0	0	4	0.11~0.45	0.25
37	dimethachlone	3	97.9	1	2	0	0.054~0.61	0.27
38	cyhalothrin	3	96.8	1	2	0	0.076~0.078	0.077
39	diethofencarb	3	95.9	2	0	1	0.019~0.066	0.037
40	thiamethoxam	3	109.8	3	0	0	0.011~0.046	0.026
41	fluopicolide	3	111.1	0	1	2	0.0085~0.074	0.031
42	diazinon	3	97.7	2	0	1	0.011~0.036	0.023

**Table 3:** Pesticide residue ranges, limit values, and toxicological data.

Pesticides	MAX Means /(mg/kg)	AVG Mean mg·kg^−1^	JMPR Data (mg/kg bw)	
			ADI	ARFD
dimethomorph	1.12	0.053	0.2	0.6
pyraclostrobin	1.17	0.031	0.03	0.05
pyrimethanil	1.85	0.035	0.2	UN
cyprodinil	0.75	0.026	0.03	UN
propiconazole	0.82	0.019	0.07	0.3
difenoconazole	0.84	0.024	0.01	0.3
quintozene	1.6	0.56	0.01	/
azoxystrobin	0.38	0.015	0.2	UN
epoxiconazole	0.82	0.017	0.008 [12]	0.023
chlorpyrifos	2.24	0.045	0.01	0.1
iprodione	0.43	0.019	0.06	0.06 [12]
phoxim	1.7	0.019	0.001	UN
tebuconazole	2.04	0.024	0.03	0.3
thifluzamide	1.75	0.026	0.02 [12]	/
metalaxyl	0.28	0.0088	0.08	0.5 [12]
fluxapyroxad	0.64	0.013	0.02	0.3
propamocarb	0.24	0.0076	0.4	2
boscalid	1.26	0.013	0.04	UN
flusilazole	0.47	0.0059	0.007	0.02
procymidone	0.39	0.0093	0.1	0.1
carbendazim	0.55	0.0083	0.03	0.1
fludioxonil	4.8	0.022	0.4	n
fluopyram	1.07	0.0084	0.01	0.5
mefentrifluconazole	0.11	0.0035	0.035 [12]	0.15
trifloxystrobin	0.26	0.0028	0.04	/
flumorph	0.16	0.0033	0.16 [11]	/
pendimethalin	0.61	0.0042	0.1	1
acetochlor	0.37	0.0043	0.01	1
atrazine	0.6	0.0042	0.02	0.1
clothianidin	0.046	0.0024	0.1	0.6
carbofuran	0.46	0.0037	0.001	0.001
fluazinam	0.075	0.0026	0.01 [12]	0.07 [12]
butachlor	0.45	0.0049	0.1 [11]	/

Note: ‘UN’ is an unnecessary acronym, indicating that an ARfD is unnecessary/indicates that the dose is not found; the Joint Meeting on Pesticide Residues (JMPR); maximum values (MAX); average values (AVGs).

## Data Availability

The original contributions presented in the study are included in the article/Supplementary Materials, further inquiries can be directed to the corresponding author.

## Supplementary Materials

The following supporting information can be downloaded at: https://www.mdpi.com/article/10.3390/foods14081381/s1, Table S1: Detection methods and recovery of GC-MS/MS; Table S2: Detection methods and recovery rate of LC-MS/MS.
