# Supplementary material for: Assessment of Pesticide Residues and Dietary Risks in Ginseng from Northeastern China
Source: Foods. 2025 Apr 17;14(8):1381. doi: 10.3390/foods14081381 (PMC12026524; doi:10.3390/foods14081381)
Supplement: Supplementary file 1 [file foods-14-01381-s001.zip › foods-3555418-supplementary.pdf]

Table S1 Detection methods and recovery of GC-MS/MS

| No. | Name | Retention Time (min) | Ion Pairs | Collision Energy (V) | Recovery (%) (RSD%) | LOQ (μg/kg) |
|---|---|---|---|---|---|---|
| 1 | Tetrachloronitrobenzene | 16.2 | 202.9/142.9,200.9/82.9 | 16、20 | 108.6(7.8) | 0.41 |
| 2 | Hexachlorobenzene | 16.77 | 283.8/213.9,283.8/248.9 | 28、16 | 88.8(3.1) | 0.13 |
| 3 | α-Hexachlorocyclohexane | 17.77 | 218.8/183,182.8/146.7 | 8、12 | 84.1(23.3) | 0.09 |
| 4 | Pentachloronitrobenzene | 18.04 | 295/237,236.9/142.9 | 20、30 | 89.6(12.5) | 0.30 |
| 5 | Terbufos | 18.07 | 230.8/175,230.8/203 | 13、5 | 82.3(19.4) | 0.10 |
| 6 | Clomazone | 18.19 | 125/89.1,125/99 | 15、15 | 92(29.8) | 0.23 |
| 7 | Pirimiphos-methyl | 18.32 | 234/110,234/126 | 10、10 | 88.1(2.3) | 0.10 |
| 8 | Atrazine | 18.57 | 200/122.1,200/132 | 8、8 | 102.4(19) | 0.04 |
| 9 | α-Hexachlorocyclohexane | 18.65 | 218.7/183,180.9/145 | 8、14 | 92.8(14.4) | 0.24 |
| 10 | Terbuthylazine | 18.74 | 214.1/104,214.1/132 | 14、8 | 83.9(1) | 0.07 |
| 11 | Heptachlor | 19.07 | 271.8/236.9,100/65 | 12、10 | 84.5(13.9) | 0.07 |
| 12 | Aldrin | 19.31 | 262.9/192.9,262.9/228 | 32、18 | 82.3(23.5) | 0.94 |
| 13 | Pentachloroaniline | 19.31 | 264.8/194,264.8/230 | 18、8 | 75.4(21.2) | 0.10 |
| 14 | Chlorpyrifos-methyl | 19.5 | 287.9/273,285.9/271 | 12、12 | 93(13.5) | 0.09 |
| 15 | Methyl Pentachlorophenyl sulfide | 19.65 | 296/263,296/281 | 15、20 | 108(4.7) | 5.51 |
| 16 | Dieldrin | 19.65 | 262.7/192.7,255/220 | 30、20 | 83.3(13.9) | 0.49 |
| 17 | Acetochlor | 19.65 | 146/117.7,131.8/117 | 8、14 | 111.5(8.2) | 1.85 |
| 18 | Tolclofos-methyl | 19.78 | 267/222,267/252 | 20、10 | 61(2.3) | 0.30 |
| 19 | Pirimiphos-methyl | 19.91 | 305.1/180.2,305.1/290.2 | 6、8 | 80.7(25.8) | 0.03 |
| 20 | Vinclozolin | 20.07 | 212/172,186.8/124 | 14、18 | 105.4(11) | 0.20 |
| 21 | β-Hexachlorocyclohexane | 20.22 | 218.7/183,180.9/145 | 8、14 | 82.4(2.3) | 0.08 |
| 22 | Parathion-methyl | 20.58 | 263/109,124.9/79 | 12、6 | 116.6(1) | 0.27 |

| | | | | | | |
|---|---|---|---|---|---|---|
| 23 | Dicofol | 20.78 | 139/111,111/74.9 | 12、12 | 84.5(14.5) | 0.72 |
| 24 | Metolachlor | 20.8 | 238.1/133.1,238.1/162.2 | 26、8 | 135.6(8.7) | 0.01 |
| 25 | δ-Hexachlorocyclohexane | 20.86 | 218.8/146.5,218.8/182.9 | 20、8 | 79.3(13.3) | 1.29 |
| 26 | Oxychlordane | 20.99 | 184.9/120.9,115/50.9 | 10、20 | 91.5(11.4) | 0.18 |
| 27 | Fenitrothion | 21.09 | 277/109,125/79 | 16、8 | 98.1(23.9) | 0.07 |
| 28 | Heptachlor epoxide | 21.43 | 352.9/263,352.9/282 | 10、12 | 117.8(3.5) | 6.28 |
| 29 | Triadimefon | 21.46 | 208/111,208/180.8 | 20、8 | 79(27.8) | 0.14 |
| 30 | Parathion | 21.63 | 290.9/109,138.9/109 | 10、5 | 69.5(25.6) | 0.06 |
| 31 | α-Endosulfan | 22.26 | 240.8/170,240.8/205.6 | 25、15 | 93.8(8.1) | 4.55 |
| 32 | trans-Chlordane | 22.38 | 372.9/264,372.9/266 | 18、18 | 85(9.2) | 6.60 |
| 33 | cis-Chlordane | 22.62 | 374.9/265.9,374.9/303 | 18、6 | 90.6(11.8) | 5.05 |
| 34 | Butachlor | 22.73 | 176.1/147.1,160.1/130.1 | 10、25 | 107.5(6) | 0.78 |
| 35 | p,p'-DDE | 22.77 | 317.8/246,246/176.1 | 20、28 | 88.4(21.5) | 0.37 |
| 36 | Dimethachlon | 23.47 | 243/187.1,187.1/152.1 | 10、10 | 107.4(24) | 0.65 |
| 37 | Procymidone | 23.51 | 283/96.1,95.9/53 | 8、16 | 76.6(18.6) | 0.53 |
| 38 | Methidathion | 23.72 | 145/58,145/85 | 12、6 | 71.9(0.6) | 0.25 |
| 39 | Phosfolan-methyl | 23.79 | 227/92,227/167.8 | 10、10 | 71.2(7.5) | 2.34 |
| 40 | Flufenoxuron | 24.14 | 388/281,388/333 | 30、15 | 96(4.6) | 4.66 |
| 41 | o,p'-DDT | 24.59 | 236.8/165,235/165.1 | 22、22 | 95.2(3.5) | 0.31 |
| 42 | Isoprothiolane | 24.79 | 189/145.1,118/90 | 10、10 | 100.1(0.9) | 1.18 |
| 43 | Nitrofen | 25.58 | 283/252.9,202/139 | 10、30 | 101.3(1.9) | 1.41 |
| 44 | p,p'-DDD | 25.93 | 235/165.1,235/199 | 20、14 | 95.2(14.4) | 0.15 |
| 45 | β-Endosulfan | 26.34 | 271.8/236.7,194.8/159 | 15、10 | 101.7(0.2) | 8.07 |
| 46 | p,p'-DDT | 26.63 | 236.8/165,235/165.1 | 22、22 | 91(9.8) | 0.36 |
| 47 | Chlorfenapyr | 26.78 | 248.9/137.1,136.9/102 | 18、12 | 83.2(4.1) | 8.61 |
| 48 | Fipronil sulfide | 26.96 | 420/255,420/351 | 20、12 | 108(1) | 5.22 |
| 49 | Fipronil | 27.53 | 368.8/214.9,366.9/212.9 | 30、28 | 111.7(1.6) | 2.40 |

| | | | | | | |
|---|---|---|---|---|---|---|
| 50 | Bifenthrin | 27.98 | 181/165.9,181/179 | 10、12 | 93.7(3) | 0.13 |
| 51 | Endosulfan sulfate | 29.34 | 273.8/238.9,271.9/236.8 | 20、20 | 103.5(7.7) | 0.98 |
| 52 | Fludioxonil | 29.42 | 248/154.1,154.1/127 | 16、6 | 71.6(2.7) | 0.56 |
| 53 | Fenpropathrin | 29.62 | 181/126.8,97.1/55.1 | 28、6 | 129.2(6) | 1.81 |
| 54 | Iprodione | 30.7 | 315.7/273,314/245 | 8、10 | 130.1(2.6) | 9.40 |
| 55 | Cyhalothrin | 32.2 | 208.1/180.9,180.9/151.9 | 8、22 | 91.6(16.6) | 0.32 |
| 56 | Permethrin | 32.5 | 183.1/153,163/91.1 | 12、12 | 87.4(12.9) | 1.34 |
| 57 | Fipronil sulfone | 32.53 | 383/213,383/255 | 32、20 | 127.3(18.4) | 1.17 |
| 58 | Etofenprox | 35.09 | 163.1/107.1,163.1/135.1 | 15、10 | 92.4(15.3) | 0.27 |
| 59 | Flucythrinate | 38.2 | 199.1/107.1,157/107.1 | 22、12 | 89.4(14.9) | 0.83 |
| 60 | Flumorph | 39.09 | 285.1/123,285.1/165.1 | 15、10 | 88.1(31.2) | 1.14 |
| 61 | Fluvalinate | 41.15 | 250/55.1,250/200 | 16、16 | 78.3(2.8) | 1.03 |

Table S2 Detection methods and recovery rate of LC-MS/MS

| NO. | pesticides | RT/min | Quant ion | Quali ion | DP/V | CE/V | $R^2$ | Recovery %( RSD %) | LOQ （μg/kg） |
|---|---|---|---|---|---|---|---|---|---|
| 62 | Cyromazine | 1.73 | 167.1/85.1 | 167.1/125.2 | 65 | 25、24 | 0.997 | 42.7 (29.1) | 0.320 |
| 63 | Methamidophos | 1.76 | 142.1/94.1 | 142.1/125.1 | 60 | 20、19 | 0.998 | 113.3 (0.6) | 0.140 |
| 64 | Acephate | 2.34 | 184.0/143.1 | 184.0/125.1 | 47 | 12、24 | 0.996 | 102 (1.9) | 0.260 |
| 65 | Propamocarb | 2.63 | 189.1/102.1 | 189.1/144.2 | 60 | 23、18 | 0.996 | 87 (16.8) | 0.016 |
| 66 | Omethoate | 2.72 | 214.1/183.0 | 214.1/155.0 | 65 | 15、19 | 0.999 | 107.1 (5.8) | 0.190 |
| 67 | Aldicarb sulfoxide | 2.99 | 207.1/132.1 | 207.1/89.1 | 51 | 9、20 | 0.996 | 122.9 (0.1) | 0.160 |
| 68 | Pymetrozine | 3.14 | 218.1/105.1 | 218.1/107.2 | 80 | 21、25 | 0.997 | 67.9 (7.3) | 1.45 |
| 69 | Aldicarb sulfone | 3.2 | 223.0/148.1 | 223.0/76.1 | 63 | 12、10 | 0.998 | 95.3 (12) | 0.210 |
| 70 | Dinotefuran | 3.23 | 203.1/113 | 203.1/87.1 | 62 | 15、20 | 0.992 | 64.9 (1.9) | 0.34 |
| 71 | Methomyl | 3.61 | 163.0/88.0 | 163.0/106.1 | 38 | 13、13 | 0.996 | 114.7 (8.6) | 0.220 |
| 72 | Thiamethoxam | 3.77 | 292.1/211.0 | 292.1/181.2 | 53 | 17、32 | 0.998 | 111.2 (2.4) | 0.061 |
| 73 | Carbendazim | 3.78 | 192.1/160.2 | 192.1/132.2 | 55 | 25、41 | 0.998 | 85.9 (2.7) | 0.018 |
| 74 | Flonicamid | 3.87 | 230.1/203 | 230.1/148.1 | 87 | 24、38 | 0.999 | 93.1 (6.3) | 0.17 |
| 75 | Chlordimeform | 3.89 | 197.0/117.1 | 197.0/152.1 | 90 | 38、25 | 0.999 | 113.8 (21.4) | 0.110 |
| 76 | Monocrotophos | 4.01 | 224.2/193.0 | 224.2/127.1 | 63 | 11、21 | 0.998 | 93.8 (2.1) | 0.120 |
| 77 | Thiabendazole | 4.37 | 202.1/175.1 | 202.1/131.2 | 88 | 34、44 | 0.997 | 60.8 (16.6) | 0.032 |
| 78 | Imidacloprid | 4.48 | 256.2/209.0 | 256.2/175.0 | 45 | 23、26 | 0.998 | 120.2 (2.1) | 0.120 |
| 79 | Clothianidin | 4.53 | 250.1/169.1 | 250.1/132.0 | 60 | 19、23 | 0.994 | 111.6 (15.3) | 0.460 |
| 80 | Dimethoate | 4.82 | 230.0/199.0 | 230.0/125.0 | 50 | 13、28 | 0.998 | 99.4 (9.4) | 0.140 |
| 81 | 3-Hydroxycarbofuran | 4.91 | 238.0/181.0 | 238.0/163.0 | 65 | 14、20 | 1.000 | 105.1 (4.4) | 0.100 |
| 82 | Acetamiprid | 4.94 | 223.1/126.0 | 223.1/99.0 | 77 | 22、51 | 0.997 | 107.8 (3) | 0.044 |
| 83 | Sulfoxaflor | 5.12 | 278.1/105.1 | 278.1/174.0 | 61 | 13、15 | 0.993 | 106.5 (6) | 0.770 |
| 84 | Cymoxanil | 5.26 | 199.2/111.0 | 199.2/128.2 | 52 | 25、13 | 0.992 | 106.2 (7) | 1.610 |
| 85 | Tricyclazole | 5.67 | 190.0/163.0 | 190.0/136.1 | 90 | 31、39 | 0.999 | 79.4 (6.8) | 0.016 |
| 86 | Phosfolan | 5.74 | 256.1/140.0 | 256.1/168.0 | 71 | 32、24 | 0.995 | 121.6 (5.9) | 0.042 |
| 87 | Aldicarb | 5.75 | 116.1/89.0 | 116.1/70.0 | 47 | 13、13 | 0.998 | 95.2 (3.7) | 0.100 |

| | | | | | | | | | |
|---|---|---|---|---|---|---|---|---|---|
| 88 | Thiacloprid | 5.79 | 253.1/99 | 253.1/126.1 | 68 | 58、30 | 0.997 | 107.1 (4.3) | 0.81 |
| 89 | Ethirimol | 6.01 | 210.3/140.2 | 210.3/182.1 | 97 | 30、27 | 0.998 | 80.3 (2.4) | 0.040 |
| 90 | Phosphamidon | 6.16 | 300.1/174.1 | 300.1/127.1 | 78 | 19、29 | 0.997 | 106.3 (3.2) | 0.031 |
| 91 | Dichlorvos | 6.35 | 221.0/79.0 | 221.0/109.0 | 70 | 23、37 | 0.999 | 84 (7.5) | 0.630 |
| 92 | Thiophanate-methyl | 6.45 | 343.0/151.1 | 343.0/311.1 | 80 | 28、15 | 0.994 | 114.1 (0.6) | 0.220 |
| 93 | Simazine | 6.46 | 202.2/132.1 | 202.2/104.1 | 80 | 26、34 | 0.999 | 94.1 (9.1) | 0.130 |
| 94 | Metsulfuron-methyl | 6.51 | 382.2/167.1 | 382.2/198.9 | 70 | 22、30 | 0.995 | 91.4 (10.2) | 0.075 |
| 95 | Carbofuran | 6.53 | 222.2/165.2 | 222.2/123.1 | 70 | 17、29 | 0.999 | 116.3 (14.4) | 0.011 |
| 96 | Fenamiphos sulfoxide | 6.68 | 320.2/233.0 | 320.2/171.1 | 95 | 33、30 | 0.996 | 95.4 (17) | 0.045 |
| 97 | Pirimicarb | 6.71 | 239.2/195.3 | 239.2/182 | 80 | 19、22 | 0.997 | 61.9 (0.6) | 0.45 |
| 98 | Carbaryl | 6.75 | 202.1/145.1 | 202.1/127.1 | 48 | 16、38 | 0.999 | 98 (1.8) | 0.098 |
| 99 | Chlorsulfuron | 6.78 | 358.1/141.1 | 358.1/167.1 | 90 | 24、24 | 0.999 | 86.5 (8.2) | 0.240 |
| 100 | Fenamiphos sulfone | 6.8 | 336.2/266.1 | 336.1/188.0 | 89 | 28、36 | 0.999 | 97.8 (5.7) | 0.027 |
| 101 | Ethametsulfuron | 7.04 | 411.2/196.0 | 411.2/168.1 | 80 | 23、42 | 0.999 | 75.5 (23.2) | 0.026 |
| 102 | Phorate sulfoxide | 7.14 | 277.1/97.0 | 277.1/171.0 | 62 | 43、20 | 0.995 | 120.6 (4.5) | 0.073 |
| 103 | Fosthiazate | 7.16 | 284.1/104.2 | 284.1/228 | 63 | 24、14 | 0.999 | 77.2 (7.4) | 0.44 |
| 104 | Imazalil | 7.23 | 297.0/159.0 | 297.0/255.0 | 88 | 31、25 | 0.997 | 77.7 (11.7) | 0.063 |
| 105 | Phorate sulfone | 7.24 | 293.0/171.1 | 293.0/143.1 | 60 | 15、25 | 0.994 | 105.5 (1.5) | 0.110 |
| 106 | Isoprocarb | 7.24 | 194.1/95.1 | 194.1/137.2 | 53 | 20、12 | 0.999 | 85.3 (24.8) | 0.081 |
| 107 | Thifluzamide | 7.26 | 321.2/183.1 | 321.2/200.1 | 95 | 34、35 | 0.996 | 93.2 (12.6) | 0.13 |
| 108 | Forchlorfenuron | 7.44 | 248.1/129.1 | 248.1/155.1 | 65 | 24、21 | 0.991 | 77 (1.7) | 0.044 |
| 109 | Metalaxyl | 7.47 | 280.1/220.2 | 280.1/192.2 | 60 | 19、24 | 1.000 | 93.1 (11.8) | 0.015 |
| 110 | Isocarbophos | 7.47 | 312.0/270.0 | 312.0/236.0 | 89 | 20、21 | 1.000 | 106.9 (15.1) | 1.140 |
| 111 | Ametryn | 7.71 | 228.2/186.1 | 228.2/96.1 | 80 | 25、35 | 0.999 | 108.2 (11.6) | 0.010 |
| 112 | Chlorantraniliprole | 7.73 | 484.2/453.0 | 484.2/286.0 | 70 | 23、18 | 0.995 | 81.3 (3.2) | 0.150 |
| 113 | Phosmet | 7.75 | 318.3/160.1 | 318.3/133.1 | 68 | 23、49 | 0.996 | 99.1 (4) | 0.064 |
| 114 | Pyrimethanil | 7.82 | 200.0/107.0 | 200.0/82.0 | 91 | 34、37 | 0.999 | 89 (7.7) | 0.130 |
| 115 | Demeton | 7.85 | 259.1/89.1 | 259.1/60.9 | 52 | 22、47 | 0.999 | 79.7 (27.5) | 0.630 |
| 116 | Terbufos sulfone | 7.92 | 321.1/171.1 | 321.1/265.0 | 62 | 16、12 | 0.994 | 115.4 (8.7) | 0.130 |

| | | | | | | | | | |
|---|---|---|---|---|---|---|---|---|---|
| 117 | Terbufos sulfoxide | 7.95 | 305.1/243.1 | 305.1/187.1 | 50 | 15、10 | 0.998 | 100.8 (0.8) | 0.340 |
| 118 | Azoxystrobin | 7.95 | 404.1/372.1 | 404.1/344.1 | 77 | 20、34 | 0.998 | 96.5 (1.3) | 0.012 |
| 119 | Diethofencarb | 7.97 | 268.2/180.1 | 268.2/226.1 | 66 | 14、26 | 0.996 | 119.2 (0.9) | 0.340 |
| 120 | Fenamidone | 8.06 | 312.2/236.1 | 312.2/264.1 | 79 | 20、15 | 0.995 | 116.3 (4.1) | 0.150 |
| 121 | Fenpropimorph | 8.18 | 304.1/130.2 | 304.1/116.2 | 110 | 34、35 | 0.997 | 74 (13.8) | 0.074 |
| 122 | Mandipropamid | 8.19 | 412.2/328.1 | 412.2/356.2 | 84 | 20、15 | 0.999 | 92.2 (8.7) | 0.160 |
| 123 | Boscalid | 8.21 | 343.2/307.1 | 343.2/140.2 | 100 | 29、27 | 0.999 | 103.9 (2.5) | 0.31 |
| 124 | Paclobutrazol | 8.22 | 294.2/70.0 | 294.2/125.1 | 71 | 55、49 | 0.999 | 92.9 (11) | 0.060 |
| 125 | Dimethomorph | 8.23 | 388.1/165.1 | 388.1/301.0 | 115 | 30、42 | 0.999 | 103.4 (2.4) | 0.130 |
| 126 | Flutolanil | 8.25 | 324.2/262.1 | 324.2/242.1 | 83 | 26、35 | 0.996 | 77.8 (12.9) | 0.016 |
| 127 | Fluxapyroxad | 8.26 | 382.0/362.1 | 382.0/342.1 | 91 | 20、30 | 0.991 | 108.6 (5.3) | 0.044 |
| 128 | Malathion | 8.26 | 330.9/127.1 | 330.9/99.0 | 65 | 17、35 | 0.998 | 113.1 (17.2) | 0.023 |
| 129 | Fluopicolide | 8.28 | 382.9/173.0 | 382.9/145.1 | 80 | 32、72 | 0.999 | 79.7 (1.4) | 0.100 |
| 130 | Myclobutanil | 8.35 | 289.1/125.0 | 289.1/69.9 | 80 | 47、38 | 0.999 | 106 (3.1) | 0.170 |
| 131 | Triazophos | 8.42 | 314.0/286.1 | 314.0/162.1 | 80 | 24、19 | 0.993 | 48.7 (16.4) | 0.055 |
| 132 | Cyproconazole | 8.44 | 292.0/125.1 | 292.0/70.1 | 73 | 44、44 | 0.997 | 126.7 (1.3) | 0.130 |
| 133 | Isazofos | 8.46 | 314.1/120.1 | 314.1/162.0 | 80 | 23、38 | 0.997 | 57.9 (8.1) | 0.026 |
| 134 | Triadimenol | 8.47 | 296.1/70.1 | 296.1/99.2 | 50 | 35、20 | 0.998 | 81.1 (20.3) | 0.150 |
| 135 | Mepanipyrim | 8.49 | 224.1/77.0 | 224.1/106.1 | 94 | 57、33 | 0.993 | 45 (8.2) | 0.050 |
| 136 | Fluopyram | 8.5 | 397.2/208.0 | 397.2/173.0 | 85 | 30、38 | 0.991 | 116.8 (3.3) | 0.018 |
| 137 | Fluquinconazole | 8.52 | 376.1/307.0 | 376.1/349.0 | 90 | 36、28 | 0.999 | 57.2 (24.4) | 0.250 |
| 138 | Fenarimol | 8.56 | 331.1/268.1 | 331.1/259.0 | 100 | 32、35 | 0.998 | 92.2 (4.3) | 0.300 |
| 139 | Cyflufenamid | 8.58 | 302.0/97.1 | 302.0/143.1 | 100 | 30、42 | 0.998 | 92.1 (16.2) | 0.140 |
| 140 | Bixafen | 8.59 | 379.9/147.1 | 379.9/175.1 | 94 | 46、27 | 0.992 | 87.6 (13.8) | 0.17 |
| 141 | Tebuconazole | 8.62 | 318.2/125.0 | 318.2/191.1 | 87 | 50、30 | 0.998 | 73.3 (3.5) | 3.810 |
| 142 | Fipronil | 8.63 | 465.1/423.1 | 465.1/107.2 | 120 | 35、35 | 0.993 | 102.1 (5) | 0.2 |
| 143 | Ethoprophos | 8.66 | 243.2/97.0 | 243.2/131.0 | 72 | 44、28 | 0.997 | 88.2 (6.4) | 0.035 |
| 144 | Flusilazole | 8.67 | 294.2/70.2 | 294.2/135.2 | 79 | 34、28 | 0.999 | 88.4 (3.3) | 1.52 |
| 145 | Cyazofamid | 8.68 | 325.1/108.0 | 325.1/261.1 | 70 | 18、14 | 0.994 | 105.6 (0.8) | 0.071 |
| 146 | Fenpyroximate | 8.69 | 332/272.1 | 332/230 | 80 | 19、26 | 0.990 | 77.6 (16.4) | 0.27 |

| | | | | | | | | | |
|---|---|---|---|---|---|---|---|---|---|
| 147 | Epoxiconazole | 8.7 | 330.2/121.1 | 330.2/123.1 | 82 | 27、23 | 0.990 | 94.7 (0.3) | 0.045 |
| 148 | Thifluzamide | 8.71 | 526.9/486.9 | 526.9/168.0 | 100 | 40、30 | 0.991 | 119.8 (3.6) | 0.380 |
| 149 | Fenbuconazole | 8.72 | 337.1/125.1 | 337.1/70.1 | 90 | 46、46 | 0.999 | 88.5 (12.5) | 0.100 |
| 150 | Diflubenzuron | 8.74 | 311.0/141.1 | 311.0/158.1 | 65 | 46、20 | 1.000 | 84.8 (15.8) | 0.310 |
| 151 | Bupirimate | 8.77 | 317.2/166.1 | 317.2/272.2 | 103 | 32、28 | 0.995 | 90.6 (11.4) | 0.038 |
| 152 | Flusilazole | 8.81 | 316.2/247.1 | 316.2/165.1 | 90 | 25、38 | 0.994 | 96 (16) | 0.041 |
| 153 | Fenamiphos | 8.81 | 304.1/217.0 | 304.1/201.9 | 83 | 31、46 | 0.998 | 80.2 (0.6) | 0.011 |
| 154 | Picoxystrobin | 8.83 | 368.2/145.1 | 368.2/205.1 | 60 | 31、13 | 0.990 | 116.9 (16.7) | 0.140 |
| 155 | Bixafen | 8.86 | 414.1/394.1 | 414.1/374.1 | 97 | 21、31 | 0.999 | 120.7 (16.7) | 0.084 |
| 156 | Cyprodinil | 8.92 | 226.2/93.1 | 226.2/108.1 | 100 | 50、34 | 0.999 | 31 (7) | 0.064 |
| 157 | Chlorbenzuron | 8.93 | 309.0/139.1 | 309.0/156.1 | 65 | 42、20 | 0.991 | 74.5 (24.3) | 0.130 |
| 158 | Kresoxim-methyl | 8.95 | 314.0/222.0 | 314.0/235.1 | 61 | 20、21 | 0.999 | 85.5 (2.6) | 0.610 |
| 159 | Isofenphos-methyl | 8.97 | 332.2/273.0 | 332.2/231.0 | 50 | 9、19 | 0.977 | 126.8 (6.7) | 2.040 |
| 160 | Sulfotep | 8.98 | 323.1/97.0 | 323.1/171.0 | 73 | 56、20 | 0.993 | 93 (9.6) | 0.026 |
| 161 | Penconazole | 8.99 | 284.2/159.0 | 284.2/173.0 | 80 | 42、27 | 0.999 | 89.1 (15.2) | 0.160 |
| 162 | Tolylfluanid | 8.99 | 364.2/238.0 | 364.2/137.1 | 40 | 20、40 | 0.991 | 101 (28.5) | 0.440 |
| 163 | Uniconazole | 9.01 | 292/69.9 | 292/124.9 | 95 | 57、46 | 0.997 | 69.6 (3.2) | 1.76 |
| 164 | Tebuconazole | 9.04 | 308.0/125.0 | 308.0/70.0 | 87 | 54、55 | 0.999 | 121.5 (14.8) | 0.480 |
| 165 | Penthiopyrad | 9.06 | 360.1/256.1 | 360.1/276.1 | 83 | 31、21 | 0.978 | 91.1 (2.6) | 0.33 |
| 166 | Fonofos | 9.09 | 247.1/109.0 | 247.1/137.1 | 63 | 25、16 | 0.998 | 91.5 (16.6) | 0.210 |
| 167 | Coumaphos | 9.11 | 363.0/226.9 | 363.0/306.9 | 100 | 36、24 | 0.995 | 52.3 (5.9) | 0.025 |
| 168 | Diazinon | 9.11 | 305.1/169.2 | 305.1/153.1 | 75 | 27、27 | 0.996 | 85.8 (1.6) | 0.025 |
| 169 | Silthiopham | 9.13 | 268.3/252.2 | 268.3/139.1 | 100 | 15、25 | 0.984 | 89.7 (8.3) | 1.26 |
| 170 | Propiconazole | 9.14 | 342.1/159.0 | 342.1/205.0 | 98 | 37、26 | 0.994 | 91.7 (18.6) | 0.089 |
| 171 | Benalaxyl | 9.14 | 326.2/294.2 | 326.2/208.1 | 79 | 16、20 | 0.997 | 111.6 (8.1) | 0.110 |
| 172 | Zoxamide | 9.14 | 336.0/187.0 | 336.0/204.1 | 78 | 30、25 | 1.000 | 85 (24.3) | 0.030 |
| 173 | Phoxim | 9.17 | 299.1/77.0 | 299.1/129.1 | 57 | 46、16 | 0.999 | 97.2 (4.2) | 0.063 |
| 174 | Pyraclostrobin | 9.17 | 382.2/194 | 382.2/163.2 | 72 | 15、34 | 0.994 | 88.9 (7.3) | 0.13 |
| 175 | Hexaconazole | 9.18 | 314.2/185.1 | 314.2/159.0 | 90 | 28、42 | 0.997 | 47.6 (22) | 1.080 |
| 176 | Mefentrifluconazole | 9.18 | 398.2/70.1 | 398.2/182.2 | 90 | 60、60 | 1.000 | 83 (19.7) | 0.44 |

| | | | | | | | | | |
|---|---|---|---|---|---|---|---|---|---|
| 177 | Pyraclostrobin | 9.19 | 388.0/194.1 | 388.0/296.1 | 60 | 17、19 | 1.000 | 84.5 (2.8) | 0.019 |
| 178 | Prochloraz | 9.22 | 376.1/308.0 | 376.1/70.1 | 65 | 17、43 | 0.992 | 92 (30.3) | 0.042 |
| 179 | Fluazinam | 9.22 | 318/141 | 318/234 | 100 | 39、23 | 0.985 | 103.6 (8.2) | 0.42 |
| 180 | Azoxystrobin | 9.23 | 413.2/145.1 | 413.2/205.0 | 61 | 35、15 | 0.999 | 80.6 (11.1) | 0.062 |
| 181 | Cyflufenamid | 9.23 | 413.2/241.1 | 413.2/295.1 | 75 | 33、21 | 0.994 | 97.1 (2.9) | 0.110 |
| 182 | Phorate | 9.24 | 261.0/75.1 | 261.0/199.1 | 49 | 20、10 | 0.996 | 83.5 (9.4) | 2.310 |
| 183 | Phosalone | 9.24 | 368.0/182.1 | 368.0/322.0 | 62 | 24、14 | 0.998 | 88.1 (8.5) | 0.088 |
| 184 | Bitertanol | 9.26 | 338.1/269.2 | 338.1/99.2 | 50 | 13、20 | 0.994 | 85.3 (11.2) | 3.200 |
| 185 | Metconazole | 9.3 | 320.2/70.1 | 320.2/125.2 | 105 | 57、57 | 0.992 | 94.3 (14.4) | 0.41 |
| 186 | Famoxadone | 9.31 | 392.2/331.2 | 392.2/238.1 | 40 | 14、24 | 0.971 | 92.8 (12.4) | 0.23 |
| 187 | Metrafenone | 9.32 | 409.2/209.1 | 409.2/227.0 | 64 | 21、30 | 0.998 | 96.3 (0.6) | 0.077 |
| 188 | Tridemorph | 9.33 | 298.3/130.2 | 298.3/98.1 | 110 | 35、38 | 0.999 | 37.9 (7.1) | 0.087 |
| 189 | Diniconazole | 9.35 | 326.1/70.1 | 326.1/159.0 | 95 | 60、42 | 0.998 | 86.3 (35.5) | 0.180 |
| 190 | Pencycuron | 9.36 | 329.2/125.1 | 329.2/218.1 | 93 | 31、22 | 1.000 | 56.5 (0.9) | 0.013 |
| 191 | Difenoconazole | 9.38 | 406.1/251.0 | 406.1/337.0 | 120 | 37、23 | 0.999 | 96.8 (5) | 0.180 |
| 192 | Cadusafos | 9.45 | 271.2/131.0 | 271.2/159.0 | 63 | 30、20 | 0.999 | 95.8 (16.1) | 0.051 |
| 193 | Amisulbrom | 9.47 | 276.1/149.2 | 276.1/176.2 | 124 | 49、51 | 0.992 | 79.5 (14.3) | 0.022 |
| 194 | Trifloxystrobin | 9.48 | 409.2/186.0 | 409.2/206.0 | 84 | 24、20 | 0.996 | 88.1 (11.4) | 0.037 |
| 195 | Triflumizole | 9.53 | 346.0/278.1 | 346.0/73.1 | 40 | 15、23 | 0.998 | 81.8 (8.6) | 0.044 |
| 196 | Tebufenozide | 9.56 | 353.3/133.1 | 353.3/105.2 | 91 | 20、48 | 0.996 | 136.9 (12.8) | 0.460 |
| 197 | Indoxacarb | 9.6 | 528/218.1 | 528/248.9 | 100 | 31、22 | 0.981 | 78.3 (9.4) | 0.68 |
| 198 | Clethodim | 9.61 | 360/164 | 360/240 | 69 | 28、21 | 0.995 | 83.8 (8.3) | 0.21 |
| 199 | Profenofos | 9.63 | 373.0/302.9 | 373.0/344.9 | 84 | 25、19 | 0.996 | 89.7 (4.6) | 0.052 |
| 200 | Fluazinam | 9.75 | 464.9/373.1 | 464.9/338.0 | 90 | 38、67 | 0.991 | 84 (12.9) | 10.0 |
| 201 | Oxathiapiprolin | 9.76 | 434.2/212 | 434.2/171 | 90 | 21、34 | 0.995 | 76 (5.2) | 0.12 |
| 202 | Cycloxydim | 9.82 | 326.1/280.1 | 326.1/180.1 | 87 | 20、30 | 0.988 | 64.7 (8.5) | 0.15 |
| 203 | Emamectin benzoate | 9.83 | 886.2/302.2 | 886.2/158.2 | 131 | 65、42 | 0.995 | 60.2 (7.4) | 1.590 |
| 204 | Oxathiapiprolin | 9.86 | 400/178 | 400/137.1 | 72 | 20、42 | 0.993 | 93.2 (0.6) | 0.26 |
| 205 | Chlorpyrifos | 9.88 | 349.9/97.0 | 349.9/198.0 | 82 | 46、28 | 0.998 | 84.2 (10.1) | 0.150 |
| 206 | Teflubenzuron | 9.89 | 381/158.1 | 381/141.1 | 117 | 21、51 | 0.982 | 99.4 (3.2) | 0.65 |

| | | | | | | | | | |
|---|---|---|---|---|---|---|---|---|---|
| 207 | Fluxapyroxad | 9.92 | 474.4/160.1 | 474.4/400.2 | 82 | 45、27 | 0.996 | 72.7 (7.2) | 2.98 |
| 208 | Pendimethalin | 9.93 | 282.1/194.1 | 282.1/212.0 | 37 | 15、25 | 0.999 | 73 (12.4) | 0.340 |
| 209 | Buprofezin | 9.94 | 306/201.1 | 306/116.1 | 70 | 17、23 | 0.997 | 102.4 (4.6) | 0.37 |
| 210 | Tolfenpyrad | 9.96 | 384.1/197 | 384.1/171.2 | 85 | 34、33 | 0.997 | 82.1 (17.2) | 0.12 |
| 211 | Hexythiazox | 10.08 | 353.2/228 | 353.2/168.2 | 84 | 22、36 | 0.999 | 88.3 (8.6) | 0.2 |
| 212 | Chlorfluazuron | 10.09 | 540.0/383.0 | 540.0/158.0 | 50 | 27、24 | 0.998 | 53.9 (16.9) | 0.190 |
| 213 | Fenpyroximate | 10.13 | 422.3/366.2 | 422.3/215.1 | 109 | 26、36 | 0.991 | 36.7 (2.7) | 0.024 |
| 214 | Pyridaben | 10.29 | 365.1/309.0 | 365.1/147.2 | 70 | 17、31 | 0.993 | 72.1 (23.4) | 0.059 |
| 215 | Abamectin | 10.45 | 895.4/751.3 | 895.4/449.3 | 120 | 60、65 | 0.992 | 86.2 (6.9) | 1.420 |
